# Molecular Diagnosis of *Taenia saginata* Tapeworm Infection in 2 Schoolchildren, Myanmar

**DOI:** 10.3201/eid2406.180217

**Published:** 2018-06

**Authors:** Eun Jeong Won, Bong-Kwang Jung, Hyemi Song, Mi-Seon Kim, Hyun-Seung Kim, Keon Hoon Lee, Min-Jae Kim, Myung Geun Shin, Jong Hee Shin, Soon-Pal Suh, Sung-Jong Hong, Woon-Mok Sohn, Thi Thi Htoon, Htay Htay Tin, Jong-Yil Chai

**Affiliations:** Chonnam National University Medical School, Gwangju, South Korea (E.J. Won, M.G. Shin, J.H. Shin, S.-P. Suh);; Korea Association of Health Promotion, Seoul, South Korea (B.-K. Jung, H. Song, M.-S. Kim, H.-S. Kim, K.-H. Lee, J.-Y. Chai);; Asan Medical Center, Seoul (M.-J. Kim); Chung-Ang University College of Medicine, Seoul (S.-J. Hong);; Gyeongsang National University School of Medicine, Jinju, South Korea (W.-M. Sohn);; National Health Laboratory, Yangon, Myanmar (T.T. Htoon, H.H. Tin)

**Keywords:** Taenia saginata, molecular diagnosis, Myanmar, children, zoonoses, tapeworms, parasites, taeniasis, helminths

## Abstract

*Taenia saginata* is the most common human tapeworm worldwide but has been unknown in Myanmar. In 2017, fecal examination in Yangon, Myanmar, revealed eggs of *Taenia* species in 2 children from a monastic school. Several proglottids expelled after medication with praziquantel were morphologically and molecularly confirmed to be *T. saginata* tapeworms.

Human taeniasis is a parasitic infection caused by tapeworm species including *Taenia saginata*, *T. solium*, and *T. asiatica* (*1*). *T. saginata* tapeworm infection is acquired through ingestion of raw or undercooked beef; pork is the infection source for *T. solium* and *T. asiatica* tapeworms (*1*). Because of differences in the life cycle, geographic distribution of these parasites can be affected by regional lifestyle, including dietary habit. Little is known about taeniasis in Myanmar. We report 2 cases of taeniasis caused by *T. saginata* tapeworms in Myanmar.

In June 2017, the Korea Association of Health Promotion, in cooperation with the National Health Laboratory, Myanmar, conducted a survey of intestinal parasitic infections near the Yangon region of Myanmar. The Institutional Review Board of the Ministry of Health and Sports, Myanmar (Ethical Review Committee no. 005117) approved the study. A total of 467 fecal samples were obtained from school-age children living in the district of Shwe Pyi Thar, Myanmar. In fecal examination using the Kato-Katz thick-smear technique, we found the eggs of *Taenia* tapeworms in 2 brothers, 8 and 10 years of age (Figure, panel A). They had never traveled out of Myanmar, and there was no possibility of consumption of imported beef. The younger boy had no specific gastrointestinal symptoms, but actively moving tapeworm segments had been found in his feces a year earlier. The older boy also had no special gastrointestinal symptoms.

**Figure:**
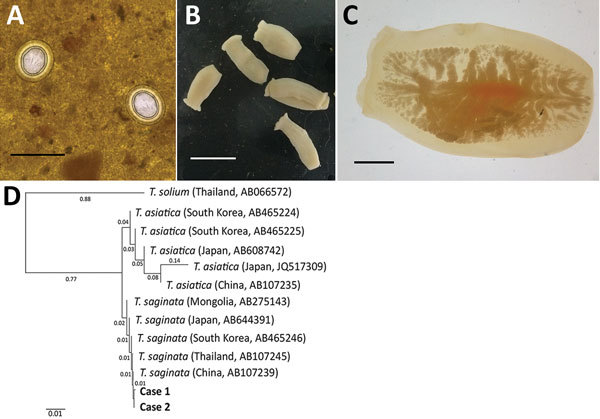
Human taeniasis caused by *Taenia saginata* tapeworms in 2 brothers 8 and 10 years of age, Yangon, Myanmar, 2017. A) Eggs of *T. saginata* from younger brother found in a Kato-Katz fecal smear. Scale bar = 50 mm. B) Proglottids from younger brother expelled after treatment with praziquantel (10 mg/kg in a single dose). Scale bar = 20 mm. C) A gravid proglottid showing >13 main lateral branches compactly. D) Phylogenetic relationships between the nucleotide sequences obtained from the 2 children (boldface; GenBank accession no. MH070609) and those of *T. saginata*, *T. asiatica*, and *T. solium* tapeworms from various countries in Asia. Scale bar indicates nucelotide substitutions per site.

The children were orphans who lived in Shan State and moved to a monastic school in the Shwe Pyi Thar area in 2015. They relied on traditional food donated from villagers, but food history related to raw beef or pork with their origin (imported or not) was unclear. However, the children reported that the Shan population usually enjoys traditional cuisine, fermented with rice and raw beef or pork. 

A sample of whole feces was collected from each child 1 day after treatment with praziquantel (10 mg/kg single oral dose). Seven tapeworm segments were recovered in feces (Figure, panel B), and >13 uterine segments filled with eggs were observed (Figure, panel C). We extracted genomic DNA from segments by using the DNeasy Blood &Tissue Kit (QIAGEN, Hilden, Germany) as recommended by the manufacturer. The mitochondrial cytochrome c oxidase 1 (*cox1*) gene was targeted in PCR amplification and sequencing. The PCR amplification was performed with primers T1F (5′-ATA TTT ACT TTA GAT CAT AAG CGG-3′) and T1R (5′-ACG AGA AAA TAT ATT AGT CAT AAA-3′), and conditions according to a previous study (*2*). Sequencing of the 502 bp *cox1* gene showed 98.8%–99.6% nt identity with *T. saginata*, but 93.8%–94.4% with *T. asiatica*, and 87.8%–88.0% with *T. solium*. These specimens were molecularly close to *T. saginata* tapeworms reported from various Asian countries but far from *T. asiatica* or *T. solium* tapeworms (Figure, panel D). Our results demonstrate that *T. saginata* tapeworms caused the taeniasis in these 2 children.

*T. saginata* tapeworms have a global distribution and are known to be endemic to Southeast Asia. Recently, epidemiologic studies of taeniasis in Myanmar have been performed; however, they focused on *T. solium* cysticercosis in pigs in Nay Pyi Taw area and seropositivity of refugee camp residents on the Thailand–Myanmar border (*3**,**4*). A report of *T. saginata* in Myanmar described only an experimental infection in animals, not human infections (*5*). Fecal examination might not be helpful in cases of taeniasis because *Taenia* spp. eggs are not differentiated morphologically. Instead, taeniasis can be diagnosed through the morphology of gravid proglottids or by immunologic or molecular techniques. In the cases we reported, a history of active movement of proglottids and >13 uterine branches in recovered segments indicated *T. saginata* rather than *T. solium* tapeworm infection, but *T. asiatica* tapeworms could not be fully ruled out. Although *T. asiatica* tapeworms can differ morphologically from *T. saginata* tapeworms (*6*), the distinctions could not always be found in each strobila; thus, molecular analyses were required to clearly distinguish them (*2**,**7*). We analyzed mitochondrial *cox1* of the *Taenia* tapeworm specimens and showed that the sequences clustered with *T. saginata* tapeworms reported from several Asia countries, but far from those of *T. asiatica* and *T. solium* tapeworms. Recently, human infections caused by hybrid infection with *T. saginata* and *T. asiatica* tapeworms in Laos were determined by sequencing the DNA polymerase delta region (*8*). Thus, for further studies, it may be useful to analyze not only the mitochondrial gene but also nuclear DNA.

Although epidemiologic surveys of *T. saginata* tapeworms have not been conducted in Myanmar, there is a strong possibility of the domestic occurrence of human taeniasis from consumption of undercooked beef or pork. Our report suggests that surveys of the prevalence and associated factors of human taeniases are urgently needed in Myanmar.
